# Establishing the consistency of a voice recognition symbol digit modalities test analogue

**DOI:** 10.1177/13524585231199321

**Published:** 2023-10-16

**Authors:** Margaret Wishart, Marina R. Everest, Sarah A. Morrow, Jonathan Rose, Lingkai Shen, Anthony Feinstein

**Affiliations:** Department of Psychiatry, University of Toronto, Toronto, ON, Canada; Department of Psychiatry, Sunnybrook Health Sciences Centre, Toronto, ON, Canada; Department of Clinical Neurological Sciences, University of Western Ontario, London, ON, Canada; London Health Sciences Centre, London, ON, Canada; Department of Clinical Neurological Sciences, University of Western Ontario, London, ON, Canada; London Health Sciences Centre, London, ON, Canada; The Edward S. Rogers Sr. Department of Electrical & Computer Engineering, University of Toronto, Toronto, ON, Canada; The Edward S. Rogers Sr. Department of Electrical & Computer Engineering, University of Toronto, Toronto, ON, Canada; Department of Psychiatry, University of Toronto, Toronto, ON, Canada; Department of Psychiatry, Sunnybrook Health Sciences Centre, Toronto, ON, Canada

**Keywords:** Multiple sclerosis, SDMT, information processing speed, cognitive assessment, automated testing

## Abstract

**Background:**

We previously demonstrated the convergent validity of a fully automated voice recognition analogue of the Symbol Digit Modalities Test (VR-SDMT) for evaluating processing speed in people with multiple sclerosis (pwMS).

**Objective/Methods:**

We aimed to replicate these results in 54 pwMS and 18 healthy controls (HCs), demonstrating the VR-SDMT’s reliability.

**Results:**

Significant correlations were found between the VR-SDMT and the traditional oral SDMT in the multiple sclerosis (MS) (*r* = −0.771, *p* < 0.001) and HC (*r* = −0.785, *p* < 0.001) groups.

**Conclusion:**

Taken collectively, our two studies demonstrate the reliability and validity of the VR-SDMT for assessing processing speed in pwMS.

## Introduction

Cognitive dysfunction can have serious consequences in the lives of people with multiple sclerosis (pwMS). The Symbol Digit Modalities Test (SDMT) evaluates information processing speed and is considered a key neuropsychological test predictive of future cognitive decline, employability, and activities of daily living in pwMS.^1^ Although the test is quick to complete, the need for a trained tester can hinder its use in clinical settings. We previously developed a fully automated voice recognition SDMT analog (VR-SDMT) and demonstrated the VR-SDMT’s convergent validity with the traditional oral SDMT in English speakers^2^ and French–Canadian speakers.^3^ We now present new data in English aiming to replicate the results from the earlier study, thereby enhancing the measure’s reliability.

## Methods

A sample of 54 pwMS and 18 healthy controls (HCs) were recruited at two tertiary care neuropsychiatry and multiple sclerosis (MS) clinics in Ontario. MS diagnosis according to the revised McDonald criteria was confirmed by a MS neurologist. All participants were 18–60 years old and provided informed consent. Exclusion criteria included prior completion of the SDMT within a year, history of traumatic brain injury, severe psychiatric illness, learning disability, or concurrent neurological disorder.

The details of the VR-SDMT analog have been described previously.^2^ The program begins by administering an eye test to confirm adequate visual acuity, which the participant must pass to advance to the test. A symbol digit code is displayed in a box with two rows: the top row has nine unique symbols and the bottom row has numbers 1–9. Below the code, there is another row with the same nine symbols in a different order, and the participant must orally report what number goes with each symbol in the row, according to the key. The program takes them through a tutorial and a practice trial to ensure they understand what needs to be done before beginning the test. The participants are instructed to proceed as quickly as possible. The test goes on for eight sequential trials, and each trial features the nine symbols arranged in a different order. The program uses Google’s online speech recognition software to time the responses. At the end, the program records the total time and mean time per trial. To control for practice effects, half of the participants completed the traditional SDMT first, and the other half completed the VR-SDMT first.

Demographic comparisons between the MS and HC groups were performed with independent samples *t*-tests or chi-square tests as appropriate. The convergent validity of the VR-SDMT and traditional SDMT was assessed with Pearson’s correlation coefficients. The relationships between test scores, Expanded Disability Status Scale (EDSS),^4^ and employment status were assessed with Spearman’s rank correlations.

## Results

Demographic and clinical data are shown in Table 1. The MS and HC groups differed only in employment rate (χ^2^ = 5.908; *p* = 0.015) (Table 1).

**Table 1.** Demographic comparisons between MS and HC participants.

	MS (*n* = 54) Mean (SD), *n* (%), median [range]	HC (*n* = 18) Mean (SD), *n* (%)	*t*-test/χ^2^	*p*
Age	41.76 (9.38)	39.17 (12.74)	*t* = 0.925	0.358
Sex (frequency, % female)	38 (70.4%)	11 (61.1%)	χ^2^ = 0.532	0.466
Education			χ^2^ = 0.533	0.465
Secondary, frequency (%)	8 (14.8%)	4 (22.2%)		
Post-secondary, frequency (%)	46 (85.2%)	14 (77.8%)		
Employment			χ^2^ = 5.908	0.015
Not working, frequency (%)	19 (35.2%)	1 (5.6%)		
Working, frequency (%)	35 (64.8%)	17 (94.4%)		
EDSS, median [range]	1.5 [0–6.5]			
Disease course				
RRMS, frequency (%)	47 (87.0%)			
SPMS, frequency (%)	6 (11.1%)			
PPMS, frequency (%)	1 (1.9%)			
Years since diagnosis	9.42 (6.79)			
Using disease-modifying drug				
Yes, frequency (%)	44 (81.5%)			
No, frequency (%)	10 (18.5%)			
Type of test done first			χ^2^ = 0.300	0.584
Traditional SDMT, frequency (%)	29 (53.7%)	11 (61.1%)		
Auto-SDMT, frequency (%)	25 (46.3%)	7 (38.9%)		

EDSS: Expanded Disability Status Scale; SDMT: Symbol Digit Modalities Test; MS: multiple sclerosis; HC: healthy control; RRMS: relapsing-remitting multiple sclerosis; SPMS: secondary progressive multiple sclerosis; PPMS: primary progressive multiple sclerosis.

**Figure 1.**
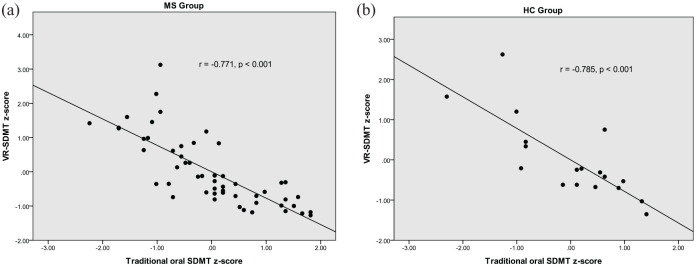
Comparison of performance on the VR-SDMT versus the traditional oral SDMT in the MS group (a) and the HC group (b).

There were significant correlations between the traditional SDMT and the VR-SDMT, for both the MS (*r* = −0.771, *p* < 0.001) and HC (*r* = −0.785, *p* < 0.001) groups. Scatterplots of these relationships are shown in Figure 1(a) and (b), respectively. The HC group had significantly faster times than the MS group on the VR-SDMT (*p* = 0.021), but the difference between groups did not reach significance on the traditional SDMT (*p* = 0.069).

In addition, EDSS scores of the MS group correlated significantly with scores on both the VR-SDMT (Spearman’s rho = 0.311, *p* = 0.022) and traditional SDMT (Spearman’s rho = −0.318, *p* = 0.019). Furthermore, unemployed participants were more likely to have slower VR-SDMT scores (Spearman’s rho = −0.268, *p* = 0.023). Employment status did not correlate with traditional SDMT scores.

## Discussion

Our data show strong convergent validity between the VR-SDMT and the traditional SDMT. We have replicated the results from our earlier studies evaluating the VR-SDMT in English^2^ and in French.^3^ In the previous study of the test in English, we found correlations between the VR-SDMT and traditional SDMT of *r* = −0.806 for the MS group and *r* = −0.629 for the HC group. In the study with French versions of the tests, we found correlations of *r* = −0.716 for the MS group and *r* = −0.623 for the HC group.

The correlations between the two versions of the tests are inverse because of how the tests are scored. The traditional SDMT is scored as the number of correct responses in 90 seconds, so better scores are higher scores. Conversely, the VR-SDMT is scored as a mean time to complete the test, so lower (i.e. faster) scores are better.

Previous studies have demonstrated correlations between SDMT scores and EDSS scores^5–7^ and SDMT scores and employability.^1^ Here, we have replicated the correlation between the EDSS, on one hand, and SDMT and VR-SDMT scores, on the other hand. We also found a correlation between VR-SDMT scores and employment status, which enhances the test’s ecological validity. The failure to find a similar result with the traditional SDMT may reflect a small sample size.

This study is limited by its modest sample size and the relatively mild level of disability. Establishing a larger normative database would allow us to update the program to calculate participants’ *z*-scores, from which impairment can be determined. Furthermore, although we have shown here the consistency of the test across different samples, indicative of good reliability, we do not yet have test–retest reliability in the same sample. As with the traditional oral SDMT version, significant dysarthria is a major barrier to the test’s use. This limitation is offset by the absence of an upper limb motor component, something which needs to be taken into account with an analogous assessment, the iPad-based Processing Speed Test.^8^

In summary, the utility of the VR-SDMT analog is that it does not need a trained tester to administer and score the test. This eliminates any inter-rater variability and makes it easy to use in a busy clinical setting. We have now shown that the test has good reliability to add to its previously demonstrated validity, making it a promising tool for clinicians to incorporate into their evaluation of processing speed in pwMS.
